# Transcriptome Sequencing Reveals a Complete Genome Sequence of *Cowpea Aphid-Borne Mosaic Virus* from Passion Fruit in Kenya

**DOI:** 10.1128/MRA.01607-18

**Published:** 2019-01-10

**Authors:** F. Munguti, S. Maina, E. N. Nyaboga, D. Kilalo, E. Kimani, M. Macharia, T. Holton

**Affiliations:** a Kenya Plant Health Inspectorate Service, Nairobi, Kenya; b Biosciences eastern and central Africa-International Livestock Research Institute, Nairobi, Kenya; c Horsham Centre, Agriculture Victoria, Horsham, Victoria, Australia; d Department of Biochemistry, University of Nairobi, Nairobi, Kenya; e Department of Plant Science and Crop Protection, University of Nairobi, Nairobi, Kenya; University of Rochester School of Medicine and Dentistry

## Abstract

Analysis of transcriptome sequencing (RNA-Seq) data revealed a complete *Cowpea aphid-borne mosaic virus* (CABMV) genome from virus-infected passion fruit in Kenya. We compared it with six complete CABMV genomes, one each from Zimbabwe and Uganda and two each from Brazil and India. The Zimbabwean isolate CABMV-Z was the closest, with 83.0% nucleotide identity.

## Abstract (precis)

Analysis of transcriptome sequencing (RNA-Seq) data revealed a complete *Cowpea aphid-borne mosaic virus* (CABMV) genome from virus-infected passion fruit in Kenya. We compared it with six complete CABMV genomes, one each from Zimbabwe and Uganda and two each from Brazil and India.

## ANNOUNCEMENT

Passion fruit (Passiflora edulis Sims) is an important horticultural crop with a significant economic and nutritional value in Africa. However, woodiness disease is the most important limiting factor in passion fruit production (1, 2). *Cowpea aphid-borne mosaic virus* (CABMV; genus Potyvirus, family Potyviridae) is the most common causal agent found to be associated with passion fruit woodiness disease in Africa (1, 2). Although CABMV has been found in Kenya (3), there is no complete genome sequence of CABMV from Kenya. In November 2014, 22 passion fruit leaf samples showing virus-like symptoms, such as mosaic, chlorosis, vein clearing, ring spots, and distorted leaves, were collected from smallholder farms in Kenya. The transcriptome sequencing (RNA-Seq) approach (4 – 8) was used to obtain a complete genome sequence of CABMV from sample 11K collected from Njoro, Nakuru County in Kenya.

With the TRIzol reagent (Sigma-Aldrich, St. Louis, MO, USA), total RNA was extracted from the 22 leaf samples preserved in silica gel, followed by RNase-free DNase (Invitrogen) treatment. Each leaf sample weighing 100 mg was ground in liquid nitrogen and then transferred to TRI reagent (800 µl) and mixed briefly, followed by incubation of the samples for 5 min at room temperature. The mixture was centrifuged at 12,000 × *g* for 10 min at 4°C, and the supernatant was transferred to a fresh microcentrifuge tube. A total of 200 µl chloroform was added and then mixed for 15 s, followed by incubation at room temperature for 15 min. The mix was centrifuged at 12,000 × *g* for 15 min at 4°C, and the supernatant was transferred to a fresh tube and then mixed with 250 µl isopropanol and 250 µl of 1.2 M NaCl/0.8 M sodium citrate. The tubes were inverted to mix, allowed to settle for 10 min at room temperature, and then centrifuged at 12,000 × *g* for 10 min at 4°C. The RNA pellet was washed by adding 1 ml of 75% ethanol, followed by brief vortexing, and was centrifuged at 8,000 × *g* for 5 min at 4°C. The ethanol was eluted, and the RNA was air-dried for 5 min at room temperature. Finally, 30 µl of nuclease-free water was added and incubated at 65°C for 10 min, followed by pipetting up and down for a final mixing. The RNA quality control was performed using a Qubit (Invitrogen), and the integrity was further confirmed using RNA screentape (TapeStation 2200; Agilent Technologies) (4 – 7). RNA-Seq libraries were prepared from the total RNA, extracted using a TruSeq stranded total RNA sample preparation kit with Ribo-Zero Plant (Illumina, San Diego, CA). The libraries were multiplexed in one lane, and a 1% PhiX v3 spike was included, and then a MiSeq instrument was used for high-throughput sequencing using a 1 × 151-cycle v2 kit (Illumina) to generate paired-end reads. The paired-end reads were subjected to quality control first by trimming using CLC Genomics Workbench version 10.1 (CLCGW; CLC bio, Qiagen, Redwood City, CA), with the quality parameter setting score limit set to 0.01, the maximum number of ambiguities set to 2, and removing any reads with less than 50 nucleotides (nt) (4 – 9). RNA-Seq reads were assembled using the *de novo* assembly method of CLCGW, with parameter settings of automatic word size, automatic bubble size, minimum contig length of 800, mismatch cost of 2, insertion cost of 3, deletion cost of 3, length fraction of 0.5, and similarity fraction of 0.9 (4 – 9). All the contigs were subjected to the CLCGW BLAST search tool and subsequently to the pairwise sequence comparison (PASC) tool and then sorted by length and examined individually. The coding regions were improved by aligning nucleotide sequences to the aligned deduced amino acid sequences using MUSCLE available in Geneious. Open reading frames (ORFs) were predicted and annotation was performed using Geneious (4 – 10). The final sequence was designated the complete coding genome based on comparison with the reference sequence used in the mapping process.

Sample 11K yielded 2,578,474 reads with a Q30 score of 98%, and after trimming, 2,438,475 reads remained, with a median length of 141 bp. The *de novo* assembly generated 478 contigs, and a single contig generated CABMV, with 14,936 reads mapping to it with a coverage of 182×. The final complete genome coding sequence length was 9,842 nt. The new sequence coded for a full polyprotein coding genome, as with other potyviruses (11). A BLAST-based search against a potyvirus genome using BLASTN 2.8.0+ (12, 13) revealed that sample 11K most resembled the Zimbabwean isolate CABMV-Z (GenBank accession number AF348210) with a 83.0% nt identity, and it shared 81% nt identity with a Ugandan isolate Serere1 (GenBank accession number KT726938) and was well within the 76% species demarcation limit for potyvirus genomes (14, 15). This study reemphasizes the importance of implementing accurate diagnostic methods to enhance plant virus control and management measures and to protect agriculture and horticultural crops from virus incursions in Kenya.

### Data availability.

The sequence described here was deposited in GenBank under accession number MH844588. Raw data were deposited in the SRA under BioSample number SAMN10352332, which is part of BioProject number PRJNA503094.
